# Delayed clearance of monkeypox virus in a patient with co infection with secondary syphilis

**DOI:** 10.1016/j.idcr.2023.e01707

**Published:** 2023-02-01

**Authors:** Almira Opardija, Geetha Sivasubramanian

**Affiliations:** Division of Infectious Diseases, Department of Internal Medicine, University of California, San Francisco, Fresno, CA, United States

**Keywords:** Monkeypox, Syphilis, Co-infection

## Abstract

Several studies from the current 2022 monkeypox (MPX) outbreak have documented co-infections with sexually transmitted infections (STIs), such as gonorrhea, chlamydia, and syphilis. We present a patient with MPX and secondary syphilis who failed to clear the MPX virus 30 days after illness onset despite a 14-day course of tecovirimat therapy.

## Introduction

The ongoing multi country monkeypox (MPX) outbreak was declared a global public health emergency by the World Health Organization in July 2022 [1]. More than 65,000 cases have been confirmed globally and 24,000 cases in the United States since the onset of the outbreak in May 2022 [2]. MPX, caused by monkeypox virus (MPXV), a zoonotic orthopox virus, is endemic to central and western Africa. Prior to this outbreak, cases outside of endemic countries were only handful and linked to animal importation or travel [3]. The current infection in this global outbreak has many atypical epidemiological and clinical features. It has emerged predominantly in men who have sex with men (MSM), often causing single or multiple painful lesions in the anogenital area without predominant prodromal symptoms [4]. Patients at risk of acquiring infection include those with multiple sex partners or recent anonymous sexual encounters. In addition, presence of genital or anal ulcerations mimics other sexually transmitted infections (STIs). Studies from this outbreak have shown 30–75% rates of co-infection with MPX and other STIs [5], [6], [7]. Tecovirimat (TPOXX), an inhibitor of orthopoxvirus envelope-wrapping protein, is a therapeutic agent recommended for selected high-risk patients infected with MPX through the Center of Disease Control (CDC) non-research Expanded Access Investigational New Drug (EA-IND) protocol [8]. Prospective placebo-controlled trials evaluating the safety and efficacy of tecovirimat in MPX treatment are lacking. Small case series have shown clinical improvement and a significant drop in viral loads within seven days of treatment [9]. It is unclear whether concurrent STIs may lead to changes in the transmission and clinical features of MPX, as well as their response to therapy. We present a patient with MPX and co-infection with secondary syphilis who, despite initial clinical response to tecovirimat therapy, failed to fully clear MPXV from the skin surface 30 days after illness onset and two days after completion of a 14-day course of tecovirimat therapy.

## Case

**Fig. 1:**
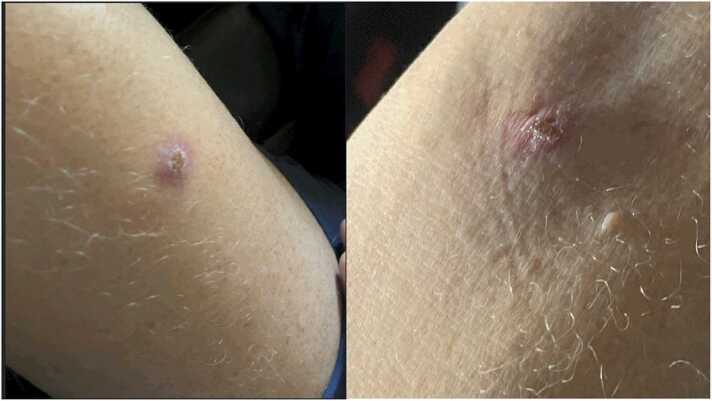
Lesions on his left thigh and right forearm initially in the course of the disease; lesions tested positive for monkeypox virus (MPXV).

A 54-year-old otherwise healthy male who was on pre-exposure prophylaxis (PrEP) with emtricitabine/tenofovir disoproxil developed symptoms of fever, headache, and myalgias three days after unprotected sexual contact with an anonymous male partner. He later developed lesions in his left thigh, right forearm, and several painful perianal lesions. He had a total of–5–6 lesions (Fig. 1). On day 14 of symptom onset, he presented to an acute care clinic because of persistent painful perianal lesions. He tested positive for MPXV by polymerase chain reaction (PCR).

The patient was referred to our institution for treatment with tecovirimat. Owing to severe painful perianal lesions, he was deemed appropriate for oral tecovirimat treatment, which was started on day 19 from the onset of his symptoms. Perianal lesions and lesions on the thigh and forearm improved dramatically. However, 6 days after the initiation of tecovirimat treatment, he started noticing a new rash all over his body, including the trunk, arms, legs, palms, and soles. These lesions were not painful and were diffuse.

**Fig. 2:**
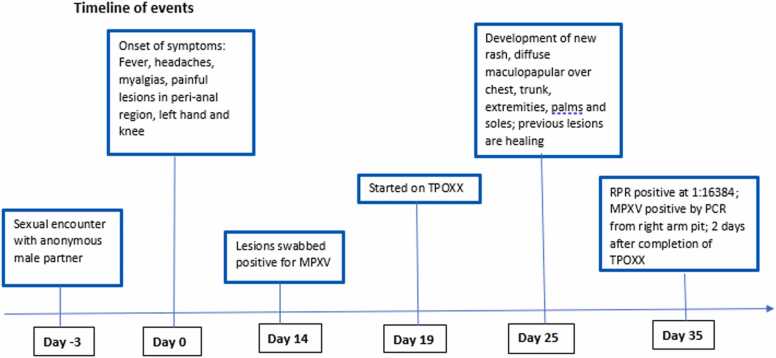
Timeline of events from the beginning of symptom-onset (day 0).

**Fig. 3:**
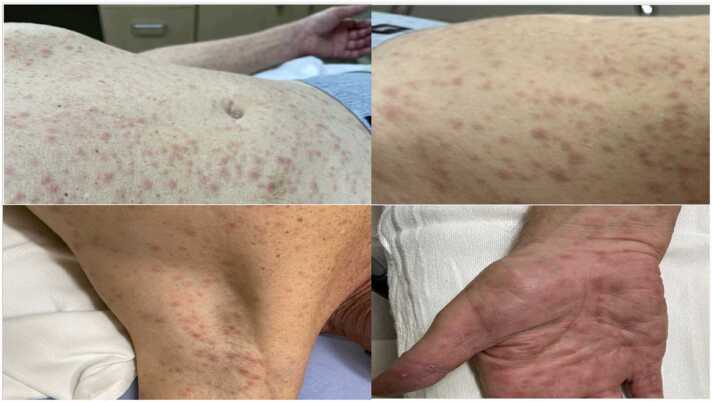
Diffuse skin lesions noted in abdomen, leg, arm pit and palm noted later in the course of the disease at the time of diagnosis of secondary syphilis. Arm pit lesion surface tested positive for MPXV by PCR.

He was advised to seek care for further testing, including testing for coinfections in his local town. He did not undergo further testing but remained on tecovirimat treatment. At completion of the 14-day treatment, he did not have any painful perianal lesions and the original lesions in his knee and finger had resolved, but he continued to have a diffuse rash throughout his body. Eventually, he returned to our emergency room two days after the completion of a 14-day course of tecovirimat and 35 days after the first symptom (Fig. 2). He was noted to have a diffuse maculopapular rash throughout his body, different from the original lesions that were present at the time of diagnosis of MPX (Fig. 3). He did not have any anogenital lesions. Multiple swabs were sent from the lesions for herpes simplex virus (HSV), varicella zoster virus (VZV), MPXV, and blood testing for syphilis. The serum rapid plasma reagin (RPR) titer was very high at 1:16384, confirming the diagnosis of secondary syphilis. The patient was treated with 2.4 million units of intramuscular benzathine penicillin. Interestingly, a lesion swabbed from his right armpit at the same time (2 days after completion of tecovirimat and 30 days after the first MPX lesion had appeared) remained positive for MPXV by PCR, albeit with a high cycle threshold of 37. After treatment with benzathine penicillin, the diffuse rash resolved in a few days, and the patient remained symptom-free. His serum RPR titer responded very well with drop to 1:256 over a period of 2 months. Multiple HIV 4th generation testing prior to and after this presentation remained negative.

## Discussion

Among persons infected with MPXV, 98% were MSM or bisexual men [5]. There is a high likelihood of sexual transmission, given the primary genital, anal, and oral mucosal lesions, possibly representing sites of inoculation and the occurrence of concomitant STIs. In a study by Thornhill et al., MPXV virus DNA was detected in the seminal fluid of 29 of 32 cases in which the seminal fluid was tested. However, it is unclear whether viral DNA can be transmitted via seminal fluid. In a study by Patel et al., *Neisseria gonorrhoeae* accounted for 21% of concomitant infections when tested, followed by Chlamydia trachomatis (11.2%), herpes simplex virus 1 or 2 (7.0%), and *Treponema pallidum* (3.7%)[6]. Few case reports have described concomitant MPX and syphilis, including MPXV with neurosyphilis and latent syphilis, in patients with HIV [10], [11], [12], [13]. Notably, one patient described in these reports with concomitant hepatitis A and steroid treatment had detectable MPXV virus by nasopharyngeal swabs at high cycle threshold values 3 weeks after completion of all treatments and healing of lesions. In our case, we noted that despite clinical improvement of the initial pox lesions, with the development of a new rash and secondary syphilis, the lesion surface continued to show detectable MPXV by PCR 30 days after initial symptom onset. At this time, the patient completed 14 days of tecovirimat treatment. It is possible that the immune dysfunction from overwhelming dissemination of treponemes in secondary syphilis interferes with the expected clearance of MPXV. This case illustrates several important aspects of this outbreak, such as diagnostic delays, the need for increased awareness about co-infections, and the possibility that co-infections may alter the mechanisms of MPX viral shedding. In our case, concomitant secondary syphilis may have impeded MPXV clearance. Further research into the immunological effects of co infection with syphilis and MPXV, as well as changes in MPX transmission and presenting features, is warranted.

## Ethical approval

The patient was made aware of our intention to present this case. In addition to written agreement/consent. Pt verbalized his understanding and agreement with our submission.

## Ethics statement

Patient anonymity was protected, and no information was identified.

## Funding statement

This study was not funded.

## Source of funding

None.

## Conflicts of Interest

The authors declare no conflict of interest.
